# Formulation of Miconazole-Loaded Chitosan–Carbopol Vesicular Gel: Optimization to In Vitro Characterization, Irritation, and Antifungal Assessment

**DOI:** 10.3390/pharmaceutics15020581

**Published:** 2023-02-08

**Authors:** Syed Sarim Imam, Sadaf Jamal Gilani, Ameeduzzafar Zafar, May Nasser Bin Jumah, Sultan Alshehri

**Affiliations:** 1 Department of Pharmaceutics, College of Pharmacy, King Saud University, Riyadh 11451, Saudi Arabia; 2 Department of Basic Health Sciences, Foundation Year of Health Colleges, Princess Nourah Bint Abdulrahman University, Riyadh 11671, Saudi Arabia; 3 Department of Pharmaceutics, College of Pharmacy, Jouf University, Sakaka 72341, Saudi Arabia; 4 Biology Department, College of Science, Princess Nourah Bint Abdulrahman University, Riyadh 11671, Saudi Arabia; 5 Environment and Biomaterial Unit, Health Sciences Research Center, Princess Nourah Bint Abdulrahman University, Riyadh 11671, Saudi Arabia; 6 Saudi Society for Applied Science, Princess Nourah Bint Abdulrahman University, Riyadh 11671, Saudi Arabia

**Keywords:** miconazole nitrate, bilosome, gel, irritation study, antifungal activity

## Abstract

Miconazole nitrate (MN) is a poorly water-soluble and antifungal drug used for fungal infections. The present research work was designed to develop topical MN-loaded bilosomes (BSs) for the improvement of therapeutic efficacy. MZBSs were prepared by using the thin-film hydration method and further optimized by using the Box–Behnken statistical design (BBD). The optimized miconazole bilosome (MZBSo) showed nano-sized vesicles, a low polydispersity index, a high entrapment efficiency, and zeta potential. Further, MZBSo was incorporated into the gel using carbopol 934P and chitosan polymers. The selected miconazole bilosome gel (MZBSoG2) demonstrated an acceptable pH (6.4 ± 0.1), viscosity (1856 ± 21 cP), and spreadability (6.6 ± 0.2 cm^2^). Compared to MZBSo (86.76 ± 3.7%), MZBSoG2 showed a significantly (*p* < 0.05) slower drug release (58.54 ± 4.1%). MZBSoG2 was found to be a non-irritant because it achieved a score of zero (standard score) in the HET-CAM test. It also exhibited significant antifungal activity compared to pure MZ against *Candida albicans* and *Aspergillus niger*. The stability study results showed no significant changes after stability testing under accelerated conditions. MZ-loaded gels could serve as effective alternative carriers for improving therapeutic efficacy.

## 1. Introduction

Candidiasis is one of the most damaging agents of infection because it can enter deeply into tissues and harm the immune system. It mostly occurs in wet, warm, and folded areas, such as the underarms and intergluteal regions. It occurs on the skin, nails, and mucous membranes [1]. This fungal infection can be treated by the topical administration of therapeutic agents, as the drug is delivered directly to the target site [2]. The efficacy of an antifungal agent depends on the ability of the drugs to penetrate through the skin layers and reach the target site. The drug should cross through the stratum corneum (outer layer of skin) and reach the epidermis and dermis layers [3,4]. Nowadays, topical fungal infections are treated with various conventional formulations, such as gel, cream, lotion, and ointment, but these formulations have drawbacks, such as poor penetration and retention resulting in poor efficacy, hypersensitivity, and sensitization reactions [3].

Miconazole nitrate (MZ) is a wide-spectrum antifungal drug that has an imidazole group, and it is used for the treatment of candidiasis. It has low systemic efficacy due to poor solubility (0.1 mg/ml) and severe hepatic alteration [5]. It functions as an antifungal agent in two different ways: by inhibiting both peroxidases and ergosterol biosynthesis, which results in the accumulation of peroxide inside the cell and, eventually, cell death [6]. Its limited water solubility has been overcome using a variety of methods, including the use of polymeric and lipid nanoparticles, as well as colloidal carrier systems. Various delivery systems such as solid lipid nanoparticles [6,7,8], transferosomal gels [4], nanocapsules [9], and organogels [10], have been reported to increase the antifungal activity of MZ. Because of limited skin penetration, topical administration presents challenges in the treatment of cutaneous disorders. Due to their capacity to bypass the skin’s natural barrier functions, lipid vesicles have received a lot of attention in recent years as topical medication carriers [6].

The use of nano-sized vesicles is a novel approach to topical delivery to the skin because of their ability to penetrate deeper layers of the skin. They absorb at boundaries or enter the stratum corneum layer by altering its barrier characteristics, which encourages them to penetrate the skin [11]. In addition, they can fluidize the stratum corneum due to the lipid bilayer present in the formulation and enhance the rate of penetration [12]. In the vesicular system, bilosomes (BSs) are novel nano-carriers composed of lipids, surfactants, and bile salts. The primary ingredients of BSs are amphiphilic bile salts that show enhanced drug penetration through intact biological membranes, including the skin and the intestine [13]. BSs are more active than other vesicles due to their high flexibility, which enhances penetration into deep skin layers [14,15]. The addition of bile salts improves the system’s colloidal stability [16], nanoscale diameter, and fluidizing action, which enhances transdermal delivery [12,17,18].

The incorporation of vesicular nano-formulations into gel systems increases drug permeability and residence time, as well as therapeutic effectiveness. Carbopol and chitosan are widely used gelling agents, and they are prominently used in the preparation of gels. Carbopol 934P is a polyacrylic cross-linked polymer and can provide efficient viscosity [19]. It is hydrophilic and has a cross-linked structure [20]. Chitosan is a natural, cationic, biocompatible, bio-adhesive, and nontoxic polymer with pronounced antibacterial properties and low immunogenicity [21]. It is commonly used as a rate-controlling and permeation enhancer for transdermal delivery [22]. Chitosan is widely used in delivery systems because its active amino and hydroxyl groups can act as sites for interaction with a variety of functional groups [23].

The present research work was designed to prepare MZ-loaded BSs by using the thin-film evaporation and hydration method. The formulations were optimized by using the Box–Behnken design by evaluating vesicle size (Y_1_) and entrapment efficiency (Y_2_). Further, the optimized miconazole bilosomes (MZBSos) were incorporated into the gel using carbopol 934P and chitosan as gelling agents. Finally, the selected miconazole bilosome gel (MZBSoG) was characterized for physiochemical parameters, in vitro release, permeation study, and in vitro and antifungal activities.

## 2. Material and Experimental

### 2.1. Material

Miconazole nitrate was procured from Unicure pharmaceutical (Noida, India). Cholesterol, Tween 80, sodium deoxycholate, chitosan (85% deacetylaton), carbopol 934P, and acetonitrile (HPLC grade) were procured from Sigma Aldrich (Bengaluru, India). A dialysis membrane (MWCO 12kda) and a Sabouraud dextrose agar medium were obtained from Hi-Media Ltd. (Mumbai, India). All other chemicals used in the experimental work were of analytical grade.

### 2.2. Experimental 

#### 2.2.1. Optimization

It is necessary to explore the independent constraints that affect the desired properties of the formulation results. MZ-loaded BSs were optimized by using a three factor–three level (low, medium, high) experimental design using the response surface methodology (Box–Behnken design, BBD). The independent constraints used in this study were cholesterol (CHO, mg), surfactant (Tween 80%), and bile salt (sodium deoxycholate, SDC). Their effects were observed on the dependent factors’ vesicle size (VS) and entrapment efficiency (EE), as shown in Table 1. Design of Experiment software (version 8.0.6, State-Ease Inc., Minneapolis, MN, USA) was employed to assess the optimization process. Three-dimensional surface response graphs indicate the relationship between independent and dependent constraints. The best-fit model was selected based on the R^2^ value. A polynomial equation was also established to examine the effect and interaction of the independent variables on the responses. 

#### 2.2.2. Formulation of Miconazole Bilosomes (MZBSs)

The MZBSs were prepared by using the film hydration and rehydration method with slight modifications to the reported method [24]. The detailed composition of the BS is shown in Table 2. The required quantities of MZ, CHO, and surfactant were dissolved in an organic solvent mixture (10 mL, chloroform: methanol, 1:1) in a round-bottomed flask. The flask was fixed to a rotatory evaporator (Rotavapor^®^ R-100, BUCHI, Flawil, Switzerland) for the removal of the organic solvent under reduced pressure. A thin film was formed on the wall of the round-bottomed flask. The flask was taken and kept for 24 h to remove traces of solvents. The formed thin film was hydrated with 10 mL of an aqueous bile salt solution. The prepared dispersion was probed and sonicated at 4 °C to reduce its size. Three cycles of sonication were performed for 30 s/cycle at an interval of 1 min. The nano-sized MZ bilosomes were stored for further characterization.

#### 2.2.3. Vesicle Characterization

The vesicle size, polydispersity index (PDI), and zeta potential of the prepared MZBSs were determined by using a size analyzer (Malvern Zetasizer, Malvern, UK). The sample was diluted 100-fold and transferred to a cuvette in order to measure the size and polydispersibility index. The same sample was also used to measure the zeta potential using an electrode-containing cuvette.

#### 2.2.4. Entrapment Efficiency (%)

The % EE of MZ in BS was analyzed by using the indirect method as reported in the literature. MZBS dispersion (5 mL) was filled into a centrifuge tube and rotated at 10,000 rpm for 30 min using a cooling centrifuge (C-24, Remi centrifuge, Remi, Mumbai, India). The supernatant was collected, and the MZ concentration of each was analyzed after dilution in methanol using a UV spectrophotometer at 271 nm [4]. The amount of MZ in each BS was calculated by using the following equation:

(1) $$\%\;\mathrm{EE}=\frac{\mathrm{Total}\;\mathrm{amount}\;\mathrm{of}\;\mathrm{MZ}-\mathrm{MZ}\;\mathrm{in}\;\mathrm{supernatant}}{\mathrm{Total}\;\mathrm{amount}\;\mathrm{of}\;\mathrm{MZ}}\times 100$$

#### 2.2.5. Development of Miconazole Bilosome Gel (MZBS Gel)

The MZBSo was converted to a gel by incorporating the sample into various concentrations of carbopol 934P (0.5–1%, *w/v*) with a fixed concentration of chitosan (CS, 0.5% *w/v*). The chitosan was dissolved in an aqueous acetic acid solution, and then carbopol 934P was incorporated. The mixture was kept overnight for complete swelling and gelling. Then, the optimized MZBS was added to the mixture with continuous stirring. Finally, triethanolamine (0.5%) and methylparaben (0.1%) were added and mixed. The pH was adjusted, and the prepared gel was stored at room temperature for further study. A neat MZ gel was also prepared using the same procedure for a comparative analysis.

#### 2.2.6. Evaluation of MZBS Gel

The prepared miconazole bilosome gels were evaluated for different parameters, such as viscosity, pH, and spreadability [25,26]. The viscosity of all MZBS gels was determined by using a Brookfield viscometer (V420001, Fungi Lab, Sant Feliu Llobregat, Spain). The study was performed in triplicate at centipoise (cP). The pH of the prepared MZBS gel was analyzed using a digital pH meter. The electrode was dipped in MZBS gel for 2 min, and the pH was measured. The spreadability was determined using the Petri plate method. The MZBS gel (1 gm) was placed on a Petri plate, and the initial diameter was noted. A second Petri plate was placed over the gel, a weight (100 gm) was placed over the second Petri plate for 30 s, and then the final diameter was noted. The spreadability was calculated using the following equation:

(2) $$\%\;\mathrm{Spreadability}\;=\frac{\mathrm{Fd}-\mathrm{Fi}\;}{\mathrm{Fi}\;}\times 100$$

where Fd is the final diameter of the gel after the application of the weight, and Fi is the initial diameter of the gel before the application of the weight. 

#### 2.2.7. Drug Content

The prepared MZBSoG (1 gm) was dissolved into methanol and sonicated using a probe sonicator (Qsonica, Newtown, CT, USA). The sample was filtered using a membrane filter (0.22 µm), and the content was analyzed using a UV spectrophotometer after an appropriate dilution.

#### 2.2.8. In Vitro Drug Release 

The in vitro release of MZ from the optimized miconazole bilosomes (MZBSos) and the optimized miconazole bilosome gel (MZBSoG2) was carried out by using a dialysis membrane. The samples (2 mg of MZ) were transferred to a test tube, and the dialysis bag was tied to the test tube and then dipped into phosphate buffer (pH 6.4) with 30% methanol as release media (250 mL) in a beaker [7]. The temperature was maintained at 37 ± 1 °C using a magnetic stirrer (VELP-Scientifica, Long Island, NY, USA). At a predetermined interval, aliquots (2 mL) were collected, and fresh release media were simultaneously added to maintain the uniform release condition. The released content was filtered, and the MZ concentration was analyzed using a UV spectrophotometer. 

#### 2.2.9. Permeation Study

A permeation study of the MZ formulations (MZBSo and MZBSoG2) was performed using a dialysis membrane. Afterward, the membrane was washed with double-distilled water. The membrane was fixed using a diffusion cell with an effective area (1 cm^2^, 20 mL receptor volume). To perform the experiment, the diffusion medium was filled into the acceptor compartment, and the temperature was stabilized. The samples were filled into the donor cell (2 mg MZ) and stirred at 50 rpm. At a predetermined time, the permeated content (1 mL) was collected from the acceptor compartment, and the same volume of fresh media was replaced to maintain uniform study conditions. The aliquot was filtered, and the MZ content was analyzed using the previously reported validated HPLC method [27]. The HPLC method was performed utilizing a mobile-phase mixture of phosphate buffer (pH 3.5) and acetonitrile (6:4) at a flow rate of 1 mL/min, with a column (C18 column, Acclaim120C18, 4.6 mm internal diameter, 2.2 µm particle size) and an injection volume of 20 µL. The flux and enhancement (ER) were calculated.

(3) $$\mathrm{ER}=\frac{\mathrm{PC}\;\mathrm{of}\;\mathrm{the}\;\mathrm{developed}\;\mathrm{formualtion}}{\mathrm{PC}\;\mathrm{of}\;\mathrm{the}\;\mathrm{pure}\;\mathrm{MZ}}$$

#### 2.2.10. In Vitro Irritation Study

An in vitro irritation study of MZBSoG2 was evaluated using the HET-CAM test. This is an alternative method to evaluate the skin irritation of a sample because animals and humans have similar blood vessels [28]. Freshly fertilized hen eggs were procured from a local poultry farm. The eggs were placed in an egg box in the upright position of the air chamber. The eggs were incubated in an incubator (Binder, NY, USA) at 37 ± 1 °C/55 ± 1% RH for 10 days. The eggs were collected from the incubator on the 10th day, and the shells were removed using forceps from the side of the air chamber. Normal saline (0.9% NaCl) was added to the egg membrane and carefully removed without damaging the CAM. The eggs were randomly distributed into three groups and treated with one drop of normal saline (Group 1; negative control; 0.9% *w/v*), 0.1 M NaOH (Group 2; positive control), and MZBSoG (Group 3). Observation was carried out for five minutes, and the score was given as per the reported scoring system. The scores for various irritations are 0–0.9 = non-irritant, 1–4.9 = mild irritant, 5–8.9 = moderate irritant, and 9–21 = severe irritant [29].

#### 2.2.11. In Vitro Antifungal Activity

The antifungal activities of the selected MZBS, MZBS gel, and pure MZ gel were evaluated on the microorganisms *C. albicans* and *A. niger* (fungal strains) using the cup-plate method [7]. The required quantity of the Sabouraud dextrose agar medium was weighed and dissolved in water. The nutrient medium was sterilized at 121 °C for 15 min in an autoclave. The nutrient medium was removed from the autoclave and allowed to cool to 60 °C under aseptic conditions. The melted nutrient medium was transferred into a Petri dish and inoculated with the tested microorganisms and mixed. The plate was allowed to solidify, and a well was made using a sterile borer. The tested samples were added to each well and kept aside for 1 h. The plates were incubated at 37 °C in an incubator (Binder, NY, USA). The zone of inhibition (ZOI) was measured at 24 h and 48 h using a graduated scale.

### 2.3. Statistical Analysis 

The study was performed in triplicate, and the data are expressed as mean ± SD. GraphPad Prism (Graph Pad Software Inc., La Jolla, CA, USA) was used for the statistical analysis. A one-way ANOVA was used for the analysis of statistical data. *p* < 0.05 was taken for a statistically significant difference. 

## 3. Results and Discussion

### 3.1. Optimization

In the present study, a total of 13 formulation compositions were obtained, with 1 center and 12 factorial points (Table 2). For both responses, viz., VS (Y_1_) and EE (Y_2_), the quadratic model was found to be significant. Based on the regression coefficient and *p*-value > 0.05, the dependent variables were found to have a significant impact on the dependent variables.

### 3.2. Effect of Variables on VS (Y_1_) 

CHO had a positive impact on the VS of the bilosomes. On increasing the CHO concentration, the VS increased accordingly. This might be due to the prevention effect of CHO on the compact packing of lipids (MZBS 3). The presence of CHOL at higher concentrations in the aqueous phase led to an increase in the VS [30]. On the contrary, the surfactant exhibited the opposite effect on the VS. An increase in the surfactant concentration led to a reduction in the VS (MZBS11). This decrease in size may be due to the higher solubility of MZ in the aqueous phase. Similarly, the third variable, bile salt, had a negative effect on the VS (MZBS7) [31].

VS = +207.14 + 47.99A − 29.32B − 9.69 C −10.48 AB +1.37AC +8.87BC + 0.40A^2^ + 1.07B^2^ + 8.60C^2^ (4)

**Figure 1:**
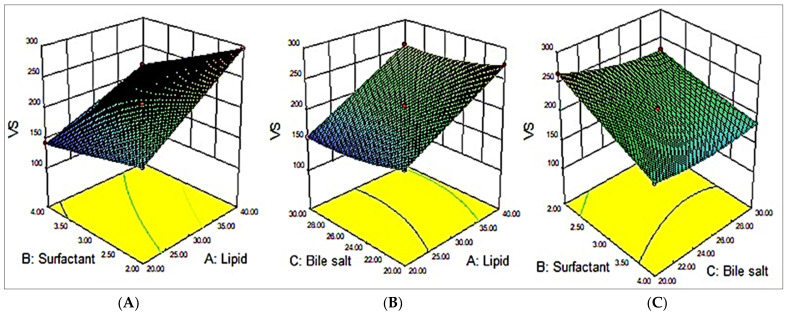
Effects of variables, namely, cholesterol (**A**), surfactant (**B**), and bile salt (**C**), on the dependent variable vesicle size (VS; Y_1_).

The effects of cholesterol, the surfactant, and bile salt were interpreted using the polynomial Equation (4) and the 3D surface response plot (Figure 1). They showed significant effects on the VS (Y_1_). Based on the outcomes achieved from the experiments and the interpretation made by BBD, it was perceived that the model terms, viz., A, B, C, AB, AC, BC, B^2^, and C^2^, showed significant (*p* < 0.05) impacts on the VS. The ANOVA of the quadratic model for each response was studied, and it was revealed that it is considerably fitted with the highest value of R^2^ (0.9598) and coefficient of variance (0.48). A reasonable agreement was observed between the predicted R^2^ (0.9505) and the adjusted R^2^ (0.9209). A summary of the statistical model and the ANOVA parameters are depicted in Table 3.

### 3.3. Effect of Variables on EE (Y_2_)

As per the polynomial equation, all three factors (A, B, and C) had a positive impact on the EE. On increasing the CHO amount, the EE increased accordingly (MZBS13). The suggested reason for the incremental action of CHO (A) on the EE might be due to the improved hydrophobicity and stiffness of the lipid bilayer membranes. This also improves permeability, stabilizes BSs, and reduces drug leakage from BSs [32]. The surfactant exhibited a positive effect on the EE; viz., on increasing the surfactant concentration, the EE of MZ increased (MZBS10). This might be due to the long alkyl chain of Tween 80, resulting in the enhanced solubility of MZ in the lipid phase and achieving enhanced entrapment efficiency. Similarly, the third variable, bile salt (C), also had a positive effect on the EE, but this effect was less prominent than that of the CHO and surfactant. SG exhibited surface-active properties by incorporating into the lipid bilayer of the BS and reducing the surface tension, as well as increasing the flexibility of the lipid membrane and, hence, increasing the EE (MZBS7) [13,33].

EE = + 76.29 +7.54A + 4.87B + 0.46 C − 0.45AB − 0.93AC +1.13B C −0.37A^2^ + 0.88B^2^ − 0.60C^2^ (5)

**Figure 2:**
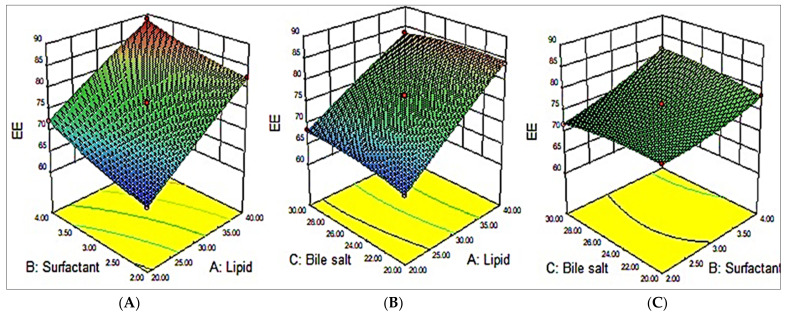
Effects of variables, namely, cholesterol (**A**), surfactant (**B**), and bile salt (**C**), on the dependent variable encapsulation efficiency (EE; Y_2_).

The polynomial equation (Equation (5)) and the 3D surface response plot (Figure 2) showed the effects of the independent variables on the EE. Based on the outcomes achieved from the experiments, it was perceived that the model terms, viz., A, B, C, AB, AC, BC, B^2^, and C^2^, had significant (*p* < 0.05) impacts on the EE. The ANOVA of the quadratic model for each response studied revealed that it is considerably fitted with the highest value of R^2^ (0.9987) and coefficient of variance (0.48). A reasonable agreement was observed between the predicted R^2^ (0.997) and the adjusted R^2^ (0.9705). A summary of the statistical model and the ANOVA parameters are depicted in Table 3.

### 3.4. Optimized Formulation 

A formulation with a composition of CHO (30 mg), surfactant (4%), and bile salt (30 mg) was selected as the optimized formulation (MZBSo). The selected optimized formulation showed a VS of 184 ± 9 nm and an EE of 83 ± 2%. This composition showed the predicted value of size to be 188 nm and an EE of 82%. The value of the desirability function should be closer to 1. A value closer to 1 is attributed to the optimal response for the factors under study [34]. For the optimized composition, the desirability function was 0.971, indicating the robustness of the optimized formulation. It was observed from the results that all the obtained values were closer to the predicted values, indicating that the optimization using BBD (RSM) is a promising approach for the optimization of MZBS.

### 3.5. Vesicle Characterization

**Figure 3:**
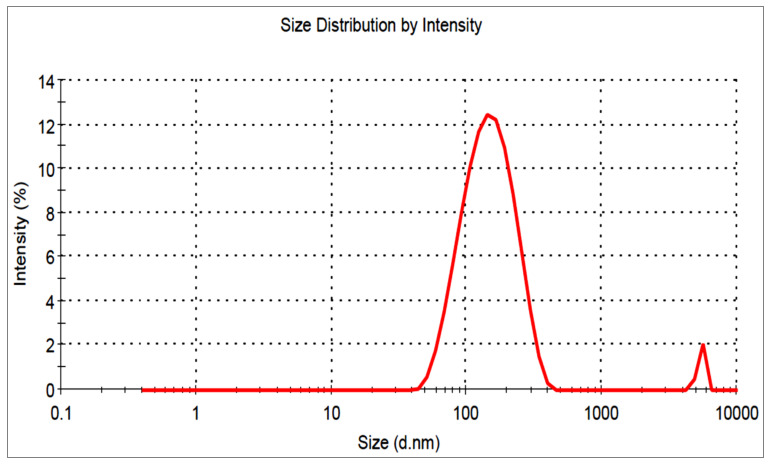
Vesicle size of the optimized miconazole bilosome (MZBSo).

The MZBSo formulation had a vesicle size of 184 ± 9.2 nm (Figure 3), and the PDI was found to be 0.078. The zeta potential of MZBSo was found to be negative (−23 mV), indicating that the vesicles were in the non-aggregate form.

### 3.6. Formulation of Miconazole Bilosome Gel (MZBSoG)

The MZBSo formulation was successfully converted into a gel by using carbopol 934P (0.5–1% *w/v*) and chitosan (0.5% *w/v*). The different characterization results are shown in Table 4. Different combinations of carbopol and chitosan were used to select the optimum range. Carbopol 934 concentrations from less than 0.5% to more than 1% with a fixed concentration of chitosan showed acceptable results. Very low and high viscosities, as well as spreadability, were not selected for the formulation design.

### 3.7. Characterization 

The viscosities of the prepared MZBSo gels (G1–G3) were found to be in the range of 1631 ± 32 cP (MZBSoG1)–2376 ± 18 cP (MZBSoG3) (Table 4). The result showed that, by increasing the carbopol concentration with a fixed CS concentration, the viscosity increased due to the ionization of the carboxylic group. A similar type of result was observed in previously reported piroxicam gel formulations [35]. The pH was determined using a digital pH meter, and the results showed no significant differences (*p* > 0.05) in the prepared compositions (Table 4). The result of the spreadability of the developed MZBS gel formulations were found to be in the order of MZBSoG1 > MZBSoG2 > MZBSoG3 (7.92 cm^2^ > 6.65 cm^2^ > 5.91 cm^2^). At higher carbopol concentrations, the spreadability decreased due to an increase in the viscosity. On the basis of viscosity and spreadability, MZBSoG2 was selected over the other formulations as the optimized formulation. The drug content of the MZBSoG formulations was determined, and the result showed a drug content between 99.34 and 99.86%. MZBSoG2 showed a pH of 6.4 ± 0.1, a viscosity of 1856 ± 21 cP, a drug content of 99.75 ± 2.72%, and a spreadability of 6.65 ± 0.21 cm^2^. 

### 3.8. In Vitro Drug Release 

**Figure 4:**
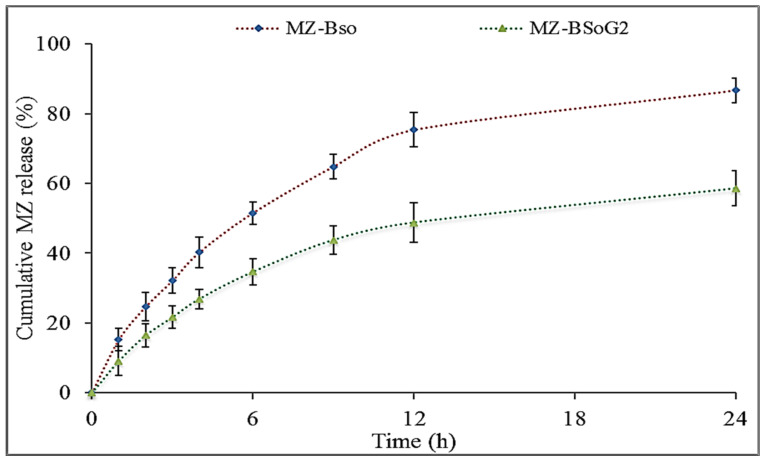
In vitro release data of miconazole bilosomes (MZBSos) and miconazole bilosome gel (MZBSoG). Study performed in triplicate, and data shown as mean ± SD. Statistical study compared between each group.

Figure 4 shows the MZ release profiles of MZBSo and MZBSoG2. The release of MZ from MZBSo was found to be 94.76 ± 3.7%, and the release of MZ from MZBSoG2 was found to be 58.54 ± 4.1%. MZBSo and MZBSoG2 showed a high release of MZ due to their nano-size and the presence of the surfactant and bile salt. The surfactant and bile salt decreased the interfacial tension, leading to an increase in the dissolution of MZ into the release media. The BS formations (MZBSo and MZBSoG2) exhibited an initial fast release in 1 h due to the presence of MZ on the BS surface. Later, a sustained and prolonged release was found due to the slow diffusion of MZ from the BS matrix [36]. MZBSoG2 showed a slower release of MZ than MZBSo because of the presence of an extra barrier of the carbopol gel matrix. The slow release of MZ from MZBSo may support transdermal delivery because an initial fast release increases permeation, and a slow release maintains the therapeutic concentration for a prolonged period of time [37]. 

### 3.9. Permeation Study

**Figure 5:**
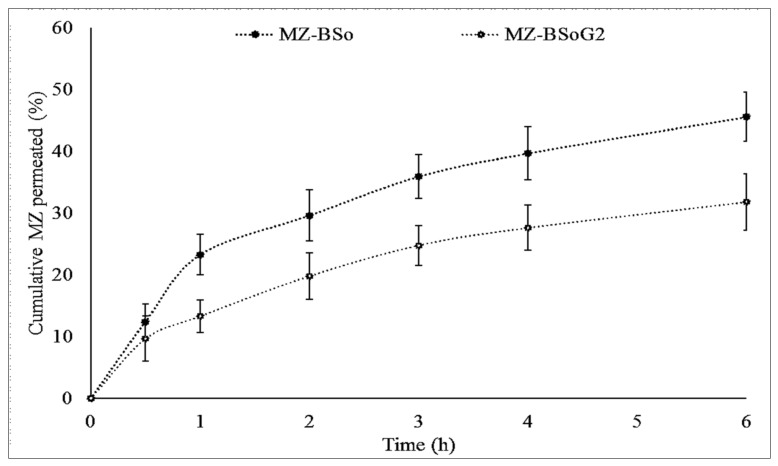
Comparative permeation study of miconazole bilosomes (MZBSos) and miconazole bilosome gel (MZBSoG2). Study performed in triplicate, and data shown as mean ± SD. Statistical study compared between each group.

The in vitro permeation of MG from MZBSo and MZBSoG2 across the egg membrane was determined, and the result is depicted graphically in Figure 5. The order of permeation of MZ was MZBSo (45.54 ± 3.8%) > MZBSoG2 (31.77 ± 4.1%). The flux of MZBSo and MZBSoG2 was also calculated and found to be 12.1 µg/cm^2^. h and 9.14 µg/cm^2^. h, respectively. MZBSoG2 exhibited significantly (*p* < 0.05) lesser permeation and flux than MZBSo due to the high viscosity and slow release of MZ from the gel matrix. The higher permeation of MZBSo and MZBSoG2 is due to their nano-size, which provides a high surface area for permeation. In addition, the presence of bile salt and surfactant also helps to increase permeation by opening the tight junction of the membrane [12]. 

### 3.10. In Vitro Irritation Test 

HET-CAM is a quick, sensitive, and economical method used to evaluate irritation. This method avoids the use of live animals for testing formulations. This test is an intermediate between in vitro and in vivo systems and does not require ethical approval [38]. The study was performed with MZBSoG2 (formulation), 0.9% NaCl (negative control), and 0.1 *M* NaOH (positive control). The prepared formulation MZBSoG2 showed a cumulative score of 0.16 (no hemorrhage of blood vessels), revealing that MZBSoG2 is a non-irritant. Similarly, the score of the negative control (0.9% NaCl) was also noted, and it also did not damage the blood vessels of CAM (zero scores). However, the positive control (0.1 *M* NaOH)-treated eggs showed a significantly (*p* < 0.001) high irritation score (18.11). A score between 9 and 21 comes under the severe irritant category (severe damage was caused to a blood vessel of CAM) and is not recommended for application [29]. From the study results, we can conclude that the prepared MZ-loaded bilosome gel is safe for application to affected areas. 

### 3.11. In Vitro Antifungal Activity 

**Figure 6:**
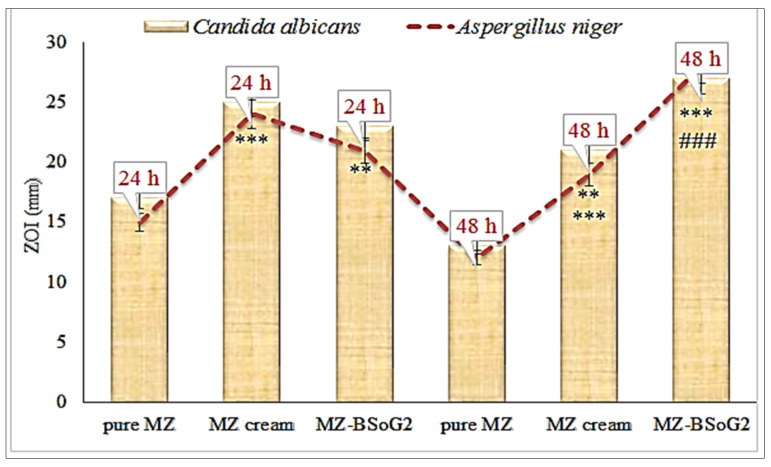
Comparative antifungal activities of pure miconazole (MZ), miconazole marketed cream (MZ cream), and optimized miconazole bilosome gel (MZBSoG2) against *C. albicans and A. niger*. Study completed in triplicate, and zones of inhibition (ZOIs) shown as mean ± SD. Statistical study compared with pure MZ. Highly significant compared to pure MZ *** (*p* < 0.001); significant compared to pure MZ ** (*p* < 0.01); Highly significant to pure MZ at 48 h ### ((*p* < 0.001).

The in vitro antifungal activities of pure MZ, a marketed cream, and MZBSoG2 were evaluated against *C. albicans* and *A. niger* using the cup-plate method. The results were noted at 24 h and 48 h, as shown in Figure 6. The pure MZ showed zones of inhibition (ZOIs) of 17 ± 1.2 mm (24 h) and 13 ± 0.9 mm (48 h) against *C. albicans.* It was also evaluated against *Aspergillus niger* and showed ZOIs of 15 ± 1.4 mm (24 h) and 12 ± 0.9 mm (48 h). The ZOI decreased significantly (*p* < 0.05) at 48 h compared to 24 h. The marketed cream displayed significantly higher ZOIs than the pure MZ at both time points against *C. albicans* and *A. niger.* The ZOIs were found to be 25 ± 1.5 mm (24 h) and 21 ± 2.2 mm (48 h) against *C. albicans.* When the ZOIs were noted against *A. niger,* they were found to be 23 ± 1.5 mm (24 h) and 19 ± 1.5 mm (48 h). The ZOI decreased significantly (*p* < 0.001) at 48 h compared to 24 h. The poor solubility and release of MZ led to a reduced effect of both the pure MZ and the marketed cream at 48 h. However, MZBSoG2 showed significantly (*p* < 0.001) high ZOIs against *C. albicans* at 24 h (23 ± 2.4 mm) and 48 h (27 ± 2.7 mm). It also showed significantly higher activity against *A. niger* at both time points. The ZOI was found to be 21 ± 1.8 mm at 24 h, and it significantly increased to 28 ± 2.6 mm at 48 h. The high antifungal activity of MZ achieved from the bilosome gel (MZBSoG2) is due to the nano-sized vesicles, the high solubility of MZ in the presence of the surfactant and bile salt, and the mucoadhesive properties of the chitosan and carbopol. Nano-sized particles can easily penetrate the walls of fungal strains due to their enhanced effective surface area for permeation. The presence of mucoadhesive polymers leads to increased penetration by helping to open the tight junction of the cell membrane and allowing for absorption. 

## 4. Conclusions

The present research was designed to develop and optimize miconazole bilosomes (MZBSs) using cholesterol, surfactant, and bile salt as independent variables. Their effects were evaluated on vesicle size and entrapment efficiency. The selected bilosomes were converted to gel formulations by using carbopol and chitosan as gelling agents, and they were further characterized for viscosity, spreadability, drug release, drug permeation, an irritation study, and antifungal activities. The optimized miconazole bilosome gel (MZBSoG2) showed the optimum viscosity, consistency, and pH and excellent spreadability. It also exhibited a sustained release. The results of a HET-CAM study revealed no signs of irritation. The antifungal study showed that MZBSoG2 exhibited significantly higher antifungal activity than pure MZ and a marketed cream against fungal strains (*C. albicans* and *A. niger*). From these findings, it can be concluded that MZ bilosome gels are potential carriers for topical/transdermal application for fungal infections.

## Figures and Tables

**Table 1:** Selected formulation variables at different levels for the optimization of miconazole bilosomes (MZBSs).

Independent Variables	Code	Low (–)	High (+)	Dependent Variables
Cholesterol (mg)	A	20	40	Vesicle size (nm; Y_1_)
Surfactant (%)	B	2	4	Entrapment efficiency (%, Y_2_)
Bile salt (mg)	C	20	30	-

**Table 2:** Composition of miconazole bilosomes (MZBSs) with their mean vesicle size and entrapment efficiency.

Formulations	Independent Variables			Dependent Variables	
	Cholesterol (mg; A)	Surfactant (%; B)	Bile Salt (mg; C)	Vesicle Size (nm; Y_1_)	Entrapment Efficiency (%; Y_2_)
MZBS 1	-	+	0	182 ± 4.9	79 ± 4.7
MZBS 2	+	-	0	295 ± 9.4	71 ± 5.3
MZBS 3	+	0	-	272 ± 12.5	81 ± 4.1
MZBS 4	0	-	-	265 ± 11.6	79 ± 4.1
MZBS 5	-	0	-	188 ± 8.2	74 ± 3.5
MZBS 6	0	-	+	227 ± 9.8	71 ± 4.1
MZBS 7	-	0	+	156 ± 6.9	68 ± 5.6
MZBS 8	0	0	0	176 ± 8.4	76 ± 4.3
MZBS 9	0	+	+	184 ± 9	83 ± 2.5
MZBS 10	0	+	-	188 ± 8.3	82 ± 2.5
MZBS 11	-	-	0	163 ± 6.3	64 ± 3.2
MZBS 12	+	0	+	256 ± 5.7	73 ± 3.9
MZBS 13	+	+	0	246 ± 10.3	87 ± 6.2

**Table 3:** Statistical model summary of selected dependent variables.

Responses	Regression Parameters	Models			Model
		Linear	2FI	Quadratic	
Vesicle size(Y_1_)	SD	9.16	5.74	0.82	Quadratic
	R²	0.9598	0.9879	0.9998	
	Adjusted R²	0.9505	0.9806	0.9996	
	Predicted R²	0.9209	0.9547	0.9989	
	% CV	-	-	0.39	
	Adeq precision	2172.51	1230.13	30.55	
	*p*-value	<0.0001	0.0059	<0.0001	
Entrapment efficiency(Y_2_)	SD	1.14	0.81	0.37	Quadratic
	R²	0.9760	0.9907	0.9987	
	Adjusted R²	0.9705	0.9851	0.9970	
	Predicted R²	0.9506	0.9555	0.9927	
	% CV			0.48	
	Adeq precision	34.72	31.31	7.02	
	*p*-value	<0.0001	0.0199	<0.0004	

**Table 4:** Characterization of different optimized miconazole bilosome gels.

Formulations	Viscosity (cP)	pH	Drug Content (%)	Spreadability (cm^2^)
MZBSoG1	1631 ± 32	6.5 ± 0.2	99.34 ± 3.13	7.92 ± 0.14
MZBSoG2	1856 ± 21	6.4 ± 0.1	99.75 ± 2.72	6.65 ± 0.21
MZBSoG3	2376 ± 18	6.5 ± 0.3	99.86 ± 3.32	5.21 ± 0.15

## Data Availability

All the data inside the manuscript.
